# Detection of Water Content in Rapeseed Leaves Using Terahertz Spectroscopy

**DOI:** 10.3390/s17122830

**Published:** 2017-12-06

**Authors:** Pengcheng Nie, Fangfang Qu, Lei Lin, Tao Dong, Yong He, Yongni Shao, Yi Zhang

**Affiliations:** 1 College of Biosystems Engineering and Food Science, Zhejiang University, Hangzhou 310058, China; npc2012@zju.edu.cn (P.N.); ffqu@zju.edu.cn (F.Q.); linlei2016@zju.edu.cn (L.L.); dt2016@zju.edu.cn (T.D.); 2 State Key Laboratory of Modern Optical Instruments, Zhejiang University, Hangzhou 310027, China; 3 Shanghai Key Lab of Modern Optical System, University of Shanghai for Science and Technology No. 516, Jungong Road, Shanghai 200093, China; ynshao@zju.edu.cn; 4 Daheng Scitech Mansion, No. 3 Suzhou Street, Haidian District, Beijing 100080, China; zhangyi@cdhcorp.com.cn

**Keywords:** terahertz spectroscopy, rapeseed leaf, water content, kernel PLS, Boosting-PLS

## Abstract

The terahertz (THz) spectra of rapeseed leaves with different water content (WC) were investigated. The transmission and absorption spectra in the range of 0.3–2 THz were measured by using THz time-domain spectroscopy. The mean transmittance and absorption coefficients were applied to analyze the change regulation of WC. In addition, the Savitzky-Golay method was performed to preprocess the spectra. Then, the partial least squares (PLS), kernel PLS (KPLS), and Boosting-PLS were conducted to establish models for predicting WC based on the processed transmission and absorption spectra. Reliable results were obtained by these three methods. KPLS generated the best prediction accuracy of WC. The prediction coefficient correlation (Rval) and root mean square error (RMSEP) of KPLS based on transmission were Rval = 0.8508, RMSEP = 0.1015, and that based on absorption were Rval = 0.8574, RMSEP = 0.1009. Results demonstrated that THz spectroscopy combined with modeling methods provided an efficient and feasible technique for detecting plant physiological information.

## 1. Introduction

Rapeseed is one of the most important oil producing and economic crops in China, accounting for more than 40% of the total area of China’s oil-bearing crops and more than 30% of the total oil production [1]. Rapeseed oil, as the main product of rape crops, is rich in oleic acid, linoleic acid and other unsaturated fatty acids. It has the effect of preventing cardiovascular disease and reducing serum cholesterol in human body [2]. Water is one of the important nutrients in photosynthesis, transpiration and nutrient transport in the process of rapeseed growth [3]. Its content in leaves is an important indicator of describing plant vitality and physiological processes [4]. Furthermore, the detection of water content plays an important role in water saving irrigation and intelligent management of the plants. 

Traditional methods for detecting water content (WC) in plant leaf are mainly drying method, distillation method, Karl Fischer method, psychrometers, pressure chambers, and gas exchange systems [5,6]. These methods are usually time-consuming. Furthermore, the validity of the data and the synchronization between different measurements cannot be guaranteed [7]. Spectroscopy methods including visible, near, mid, short-wave, thermal infrared, hyperspectral images, are applied as adequate analytical tools for leaf water detection. However, these methods usually need to extract water sensitive characteristic spectrum. Terahertz (terahertz radiation or T-ray, THz) as an advanced technology has received more and more attention in the field of biological sciences [8,9]. One of the unique properties of THz is that water has a strong absorption of electromagnetic waves in the terahertz region [10,11]. This property enables effective monitoring and analysis of the WC in agricultural products [12]. THz signal attenuation by water makes the use of THz radiation a sensitive non-contact probe of hydration [13]. Hence, THz radiation has enormous potential to detect leaf WC [14]. Gente et al. [12,15] applied the absorption of microwave radiation at 35 GHz inside the crop plants to implement the method of non-destructive and contactless measurements for the WC. Born et al. [16] measured the WC in the main-vein of silver fir (Abies alba) seedlings by THz transmission spectrum with frequencies between 0.1 and 1 THz. Hadjiloucas et al. [17] detected the THz transmittance (0.1–0.5 THz) for the Fatsia japonica and the Phormium tenax leaves under various conditions. Castrocamus et al. [18] compared the water retention capacity of the leaves of Arabidopsis plants growing in two substrates. Ogawa et al. [19] detected the moisture change of a Hedera helix leaf by a transillumination THz imaging system. All these previous works have successfully demonstrated that the THz sensor has great potential for water detection of plant leaves. These studies evaluated the spectral responses to the change of leaf water status and showed the potential of THz spectroscopy for detecting leaf water content. 

In this paper, the THz sensor was used to detect the WC of rapeseed leaves. Two different varieties of rapeseed were collected as samples in the experiment. The fresh leaves were detached from the rape plants and placed in the laboratory environment. The leaf WC and THz spectra were continuously monitored during the process of leaf water dehydration. The responses of time-domain and frequency-domain spectra to the change of leaf WC were analyzed. In addition, the modeling methods including Partial Least Squares (PLS), Kernel PLS (KPLS) [20], Boosting-PLS [21] were performed to investigate the THz spectral data and WC of rapeseed leaves. The THz transmission and absorption spectra in the range of 0.3–2 THz were obtained to establish the models for quantitatively evaluating the WC in rapeseed leaves. 

## 2. Materials and Methods

### 2.1. Experimental Setup

The THz spectrometer, China Instrument Program-Time Domain spectrometer (CIP-TDS), Inc. (Beijing, China), was used in the experiment. Its transmission mode was applied for spectral collection of the tested samples. The Ti-sapphire femtosecond laser was split into a pump beam and a probe beam by a polarization beam splitter. The pump beam was incident on the THz emission crystal to generate THz pulses. The THz pulses were focused on the detection crystal by two sets of off-axis parabolic mirrors. The probe beam was used to gate the detector and measure the instantaneous THz electric field. A delay stage was used to offset the pump and probe beams [22]. The center wavelength was 800 nm, the pulse width was 100 fs, the repetition frequency was 80 MHz, and the bandwidth was about 0.1 to 3.5 THz. The pulse light, collimated by the mirror, was focused on the surface of the test sample. After reflected by the silicon (Si), the probe light collided with the THz light carrying the sample information [23]. After the collinear light passing through the probing crystals of zinc telluride (ZnTe), the corresponding THz spectrum of the tested sample was obtained by detecting the change of the polarization state [24]. To avoid the effect of moisture in the air on the test results, the sample bin was filled with nitrogen and the humidity inside the system was less than 5%. The laboratory temperature was 294 K and the humidity was below 20%.

### 2.2. Sample Preparation

Two varieties of rapeseed leaves were collected in the experiment. They were New Oil and Long Oil, respectively. The rapeseed plants were grown in the experimental farm of Zhejiang University, China. Two weeks before the experiment, the plants were transplanted to a greenhouse. The temperature was 24 °C and the humidity was 65%. Each kind of rapeseed was transplanted for 4 pots, and there were 3 plants in each pot. Moderate water was irrigated according to the status of the rapeseed plants. In each pot, one fresh leaf from the same leaf position was picked. Thus, 8 leaves were taken, and there were 4 leaves in each of the two species of rape. These leaves were similar in size, grew well with no diseases and pests. The leaves were taken off from the plants and then placed in laboratory. The spectra and leaf weights were measured every 30 min during the natural evaporation of leaf moisture, and the measurements were continuously repeated 10 times. Hence, a total of 80 sets of data were generated. The experiment was conducted to test the spectral response of THz to different degree of leaf WC, and quantitatively predict WC by establishing models based on the THz spectra.

### 2.3. Data Acquisition

#### 2.3.1. THz Spectra

The THz time-domain spectra of the reference and leaf samples were measured by using transmission mode of the THz sensor. On each leaf, a circular mark with a diameter of 1 cm at the center of the leaf was drawn to fix the position for spectral scanning. The centre of the circle was selected as the spectral scanning point, which was located at the mesophyll part to avoid the veins. At each scanning point of a leaf, multiple scans were accumulated to take the mean spectrum as the final spectrum of the leaf sample. The time domain spectra were transformed into the frequency domain spectra by Fast Fourier Transform (FFT). According to the models proposed by Duvillaret and Timothy [25,26], the macroscopic optical properties of the measured sample can be expressed by the complex refractive index $\tilde{n}$:

(1) $$\tilde{n}(\omega )=n(\omega )-jk(\omega )$$

n(ω) is the real refractive index of the sample. It describes the dispersion of the sample. k(ω) is the extinction coefficient. It describes the absorption characteristics of the sample. j is the imaginary part. ω=2πf, and f is the frequency. The reference spectrum $E_{ref}(\omega )$ is the transmit frequency domain waveform received by the detector directly. It can be expressed as:

(2) $$E_{ref}(\omega )=E_{0}(\omega )\exp(-j\omega t+j\frac{\omega }{c}g)$$

$E_{0}(\omega )$ is the emitted frequency domain spectrum. g is the distance that the terahertz pulse travels in free space. c is the speed of light. The signal spectrum $E_{sample}(\omega )$ through the sample can be expressed as:

(3) $$\begin{array}{c} E_{sampe}(\omega )=\frac{1}{n(\omega )+1}\cdot \frac{2n(\omega )}{n(\omega )+1}\cdot E_{0}(\omega )\cdot \exp(-\frac{k(\omega )\omega }{c}d)\cdot \\ \exp[-j\omega t+i\frac{\omega }{c}(g-d)+j\frac{n(\omega )\omega }{c}d]\; \end{array}$$

d is the thickness of the sample. 1/(n(ω)+1) is the transmission coefficient of the terahertz pulse that enters the sample. 2n(ω)/(n(ω)+1) is the transmission coefficient of the terahertz pulse that exit from the sample. Hence, the transmission spectra and absorption spectra of the sample can be expressed as follows:

The transmittance T(ω):

(4) $$T(\omega )=\frac{E_{sample}(\omega )}{E_{ref}(\omega )}=\frac{4n(\omega )}{{\left[n(\omega )+1\right]}^{2}}\cdot E_{0}(\omega )\cdot \exp[j(\frac{\omega n(\omega )-\omega }{c}-\frac{k(\omega )\omega }{c})d]$$

The absorption coefficient α(ω):

(5) $$\alpha (\omega )=\frac{2k(\omega )\omega }{c}$$

#### 2.3.2. Leaf Water Content

The weights of the leaves were measured with an electronic scale. The accuracy of the electronic scale was 0.0001 g. The thickness of the leaves was measured with a vernier caliper. The accuracy of the vernier caliper was 0.01 mm. The thickness was measured at the approximately same points as that of the THz spectra. After 10 times of measurements of the leaf spectra and weights, the leaves were placed in the oven drying at 100 °C for 1 h, then drying at 60 °C until the leaf weight stay invariant. To evaluate the change of leaf water content during the process of leaf water evaporation, the weight measurements were converted into WC values using Equation (6) [27].

(6) $$WC=\frac{W_{time}-W_{dry}}{W_{fresh}}\times 100\%$$

The weight of the fresh leaf was set as the denominator in Equation (6), which remained unchanged. Hence, unified standard was used to evaluate the change of leaf water content between two successive measurements. The weights of the leaves were monitored over time, and a total of 10 times of measurements were conducted. $W_{fresh}$ was the weight of the fresh leaf. It was taken by the first measurement of the weight. $W_{time}$ was the weight of the leaf measured over time. $W_{dry}$ was the weight of the dry leaf. It was taken by the last measurement after dried in oven.

### 2.4. Modeling Methods

#### 2.4.1. PLS Method

PLS regression is based on latent variables. The matrix X(n×m) is the input spectral data. Y(n×1) is the output measured parameter. n is the number of samples and m is the number of variables. X and Y are decomposed by PLS simultaneously, and the maximal covariance between two matrices are identified. In particular, PLS is applied to find the best correlation between X and Y. It is a combination of multiple linear regression, canonical correlation analysis and principal component analysis. The model is formulated as follows:

(7) $$X=TP^{T}+E_{X}=\sum_{k=1}^{f}t_{k}p_{k}{}^{T}+E_{X}$$

(8) $$Y=UQ^{T}+E_{Y}=\sum_{k=1}^{f}u_{k}q_{k}{}^{T}+E_{Y}$$

where f is the number of principal factor. $t_{k}(n\times 1)$ is the score of the k-th principal factor of X. $p_{k}(1\times m)$ is the loading matrix of the k-th principal factor of X. $u_{k}(n\times 1)$ is the score of the k-th principal factor of Y. $q_{k}(1\times p)$ is the loading matrix of the k-th principal factor of Y. T and U are the score matrices. P and Q are the loading matrices. P is evaluated as the covariance between X and Y, and Q is evaluated as the covariance between Y and U. $E_{X}$ and $E_{Y}$ are the residual matrices. 

The linear regression of T and U is conducted as follows:

(9) U=TB

(10) $$B={\left(T^{T}T\right)}^{-1}T^{T}Y$$

where B is the matrix of regression coefficient. The score matrix $T_{test}$ of the tested sample $X_{test}$ can be obtained by P. The prediction value of the tested sample is calculated according to Equation (11):

(11) $$Y_{test}=T_{test}BQ$$

#### 2.4.2. KPLS Method 

KPLS is as simple as the standard PLS. It can handle a wide range of nonlinearities by different kinds of kernel functions and their changeable parameters. X(n×m) and Y(n×1) are the input and output data of the calibration set respectively. n is the number of samples and m is the number of variables. f is the maximum principal factor. With specified type of kernel function, the KPLS algorithm is formulated as follows:

Step. 1. Calculating the kernel matrix K(n×n) of X by the kernel function.
Step. 2. Centering the kernel matrix K by Equation (12):
  (12) $$\tilde{K}=(I-\frac{1}{n}ll^{T})K(I-\frac{1}{n}ll^{T})$$
  where I is the identity matrix, l is the column vector of n-dimension and the element values are 1.
Step. 3. Initializing the variable u. $t=\tilde{K}u,\;t=t/\left\|t\right\|$, $c=Y^{T}t$, u=Yc,u=u/‖u‖. Computing these formulas iteratively until the algorithm converge.
Step. 4. $\tilde{K}=(I-tt^{T})\tilde{K}(I-tt^{T}),Y=Y-tt^{T}Y$, repeating Step 3 until all f vectors of u and t are obtained.
Step. 5. Calculating the prediction values of the calibration set samples. $\hat{Y}=\tilde{K}U{\left(T^{T}\tilde{K}U\right)}^{-1}T^{T}Y$, where $T=[t_{1},t_{2},\cdots ,t_{f}]$, $U=[u_{1},u_{2},\cdots ,u_{f}]$.
Step. 6. For the validation set $X_{test}$(p×m, p is the number of samples and m is the number of variables), calculating its kernel matrix $K_{test}$ and then centering $K_{test}$ by Equation (13).
  (13) $${\tilde{K}}_{test}=(K_{test}-\frac{1}{n}ll^{T}K)(I-\frac{1}{n}ll^{T})$$
Step. 7. Calculating the prediction values of the validation set samples. $\hat{Y}=\tilde{K}U{\left(T^{T}\tilde{K}U\right)}^{-1}T^{T}Y$.

#### 2.4.3. Boosting-PLS Method

The boosting regression algorithm was first developed by Freund and Schapire [11,12], and then was extended and improved by Drucker [13] to widen its range of application for practical problems. In Boosting-PLS, the boosting strategy is applied to generate the ensemble framework and PLS is applied to establish the base models during each iteration. The method of Boosting-PLS is described as:

Step. 1. Normalizing the sample weights $\omega_{i}^{\left(1\right)}=1/n(i=1,2,\cdots n)$, where n is the number of samples. Initializing the initial number of iterations T(t=1,2,⋯,T).
Step. 2. Calculating the sampling probability of each sample in the original calibration set. $P_{i}^{\left(t\right)}=\omega_{i}^{\left(t\right)}/\sum_{j=1}^{N}\omega_{j}^{\left(t\right)}$. Using the roulette method to pick n samples with replacement from the original training set.
Step. 3. Establishing the PLS base model $h_{t}$ with the n samples picked out by Step 2. Putting all the training samples into $h_{t}$. Calculating the prediction error of each sample.
  (14) $$L_{i}^{\left(t\right)}=\frac{\left|{\overset{⌢}{y}}_{i}^{\left(t\right)}-y_{i}\right|}{\max\left|{\overset{⌢}{y}}_{i}^{\left(t\right)}-y_{i}\right|},i=1,2,\cdots ,N$$
  where ${\overset{⌢}{y}}_{i}^{\left(t\right)}(i=1,2,\cdots ,N)$ is the predicted value, $y_{i}$ is the measured value.
Step. 4. Calculating the sum of the weighted error ${\bar{L}}_{t}=\sum_{i=1}^{N}L_{i}p_{i}^{\left(t\right)}$ in the *t*-th iteration
Step. 5. Calculating the indicator $\beta_{t}={\bar{L}}_{t}/(1-{\bar{L}}_{t})$. Updating the new weight $\omega_{i}^{\left(t+1\right)}=\omega_{i}^{\left(t\right)}\beta_{t}^{\left[1-L_{i}^{\left(t\right)}\right]}$ of each sample.
Step. 6. Repeating Steps 2 to Steps 5 until t=T.

For one testing sample, a total of T prediction results are obtained by the T base models. The final prediction result of this sample is obtained by the combinatorial calculation of the T prediction results.

## 3. Results and Discussion

### 3.1. Spectra of Rapeseed Leaves

**Figure 1:**
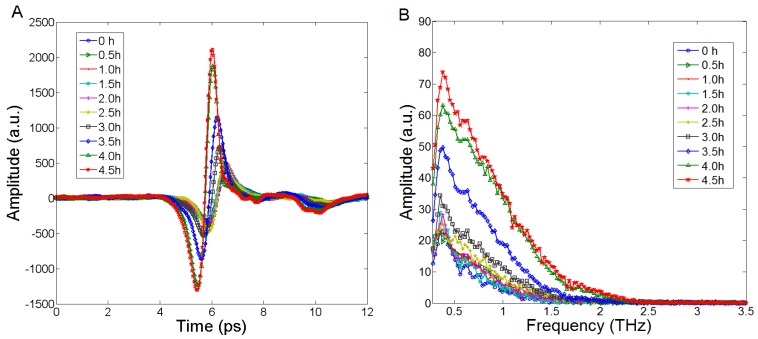
The THz spectra of rapeseed leaf. (**A**) the time domain spectra, (**B**) the frequency domain spectra.

To reveal the spectral variation over time in the experiment, the THz time domain and frequency domain spectra of one rapeseed leaf obtained from different detecting time were depicted (the rest 7 leaves showed the similar spectral variation characteristics with the change of WC). Figure 1A showed the time-domain spectra. The amplitude and time delay changes over the sample drying time were obvious. During the detection process, the amplitude of time domain spectrum increased gradually, and the time delay advanced gradually. Especially, the change of spectral amplitude was probably caused by the change of leaf WC. The change of time delay was probably caused by the change of the refractive index of the tested leaf. Figure 1B depicted the frequency domain spectra that were transformed from the time domain spectra by FFT. The frequency range was 0.1–3.5 THz, and the resolution was 25.5 GHz. Similarly, over time the spectral amplitude increased gradually. The spectral information was concentrated in the frequency ranges of 0.3–2 THz. The spectra of leaf samples at higher frequencies (above 2.0 THz) produced lower signal-to-noise ratio (SNR) due to the limitation of dynamic range of the THz spectrometer. The thickness of leaves decreased gradually over time. As demonstrated by Seelig et al. [28], the thickness appeared to decrease substantially as a result of leaf dehydration. Hence, leaf dehydration was the main factor that caused spectral variation in this experiment.

**Figure 2:**
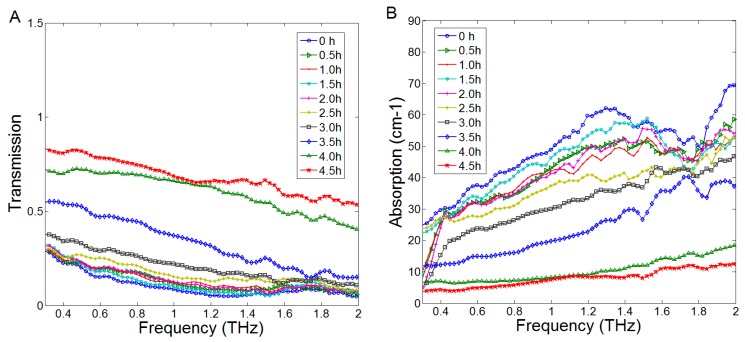
The THz spectra of one rapeseed leaf in the range of 0.3–2 THz. (**A**) Transmission spectra, (**B**) Absorption spectra.

The transmission and absorption coefficients were calculated using Equations (1) and (2), respectively. These two kinds of spectra (0.3–2 THz) of the leaf measured at 10 different detecting time were shown in Figure 2. There were no obvious absorption peaks in the spectra. The characteristics of the THz transmission and absorption spectra varied greatly with the dehydration of leaf water. As time went by, the mean transmission coefficients decreased and the mean absorption coefficients increased during the experimental process. The reason was that the moisture evaporation reduced the leaf WC. Due to the property of water absorption of THz radiation, the decrease of leaf WC led to higher transmission and lower absorption. 

### 3.2. Analysis of Leaf Water Content and THz Spectra

**Figure 3:**
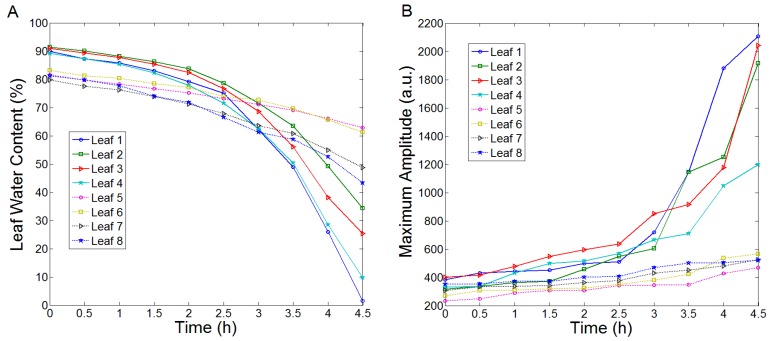

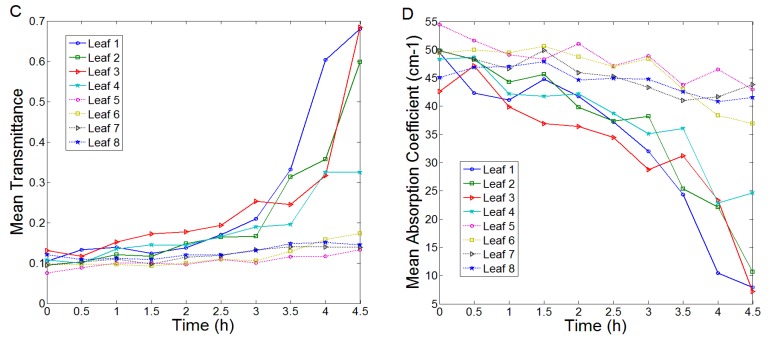
The change of WC and the corresponding THz spectral data of the rapeseed leaves. (**A**) Leaf WC, (**B**) Maximum amplitude of time domain spectra, (**C**) Mean transmittance in the range of 0.3–2 THz, (**D**) Mean absorption coefficients in the range of 0.3–2 THz.

To further explore the relationship between the leaf WC and the corresponding THz spectra, the maximum amplitude of the time domain, the mean transmittance and absorption coefficient in the range of 0.3–2 THz were used to reveal the change of WC. The results were shown in Figure 3. Leaf 1, leaf 2, leaf 3 and leaf 4 belonged to the rapeseed species of New Oil and the rest four leaves belonged to that of Long Oil. Figure 3A showed the trend of change of leaf WC during the experimental process. The change rate and change range of WC of these two species of leaves were different. Generally, the change rate of New Oil rapeseed leaves was faster than that of Long Oil rapeseed leaves. Furthermore, the change range of the former was greater than that of the latter. The possible reason could be the surface colloid of Long Oil leaves was thicker than that of New Oil leaves. It led to better water retention of Long Oil leaves, and it could slow down the dehydration rate of water. Figure 3B–D depicted the maximum amplitude of time domain spectra, the mean transmittance and mean absorption coefficients in the range of 0.3–2 THz, respectively. As can be seen, the change rules of the maximum amplitude and the mean transmittance were contrary to that of leaf WC. The maximum amplitude and mean transmittance were negatively correlated with WC. The change rule of mean absorption coefficients was approximately consistent with that of WC, and there was a positive correlation between the two. The results implied that the THz spectra could be effectively applied to reflect and evaluate the WC of leaves. 

### 3.3. Predicting Water Content by Modeling Methods

In this paper, after processed by Savitzky-Golay smooth, the transmission and absorption spectra in the range of 0.3–2 THz were applied for establishing the models. The predictive ability and stability of the models were evaluated by 4 parameters, including the correlation coefficient of calibration (Rcal), the correlation coefficient of validation (Rval), the root mean square error of calibration (RMSEC) and the root mean square error of validation (RMSEP). A good model should have higher Rcal and Rval, lower RMSEC and RMSEP. Since the ranges of WC of the two varieties of rapeseed leaves were overlapped, the samples could be merged to establish the models. The 80 samples were divided into two groups. There were 50 samples in the calibration set, and the rest 30 samples were in the validation sets. The calibration set was used for training and establishing the models, and the validation set was used for predicting the samples and testing the models. Table 1 listed the statistical results of each sample set. The division results of the sample sets varied with the spectral types. The ranges of WC of the validation sets were included in that of the calibration sets. The mean value and the standard deviation of these two kinds of samples sets were numerically close to each other. It implied that the division results were reasonable and they were compliant with modeling standards.

#### 3.3.1. Predicting Leaf Water Content with PLS Model

**Figure 4:**
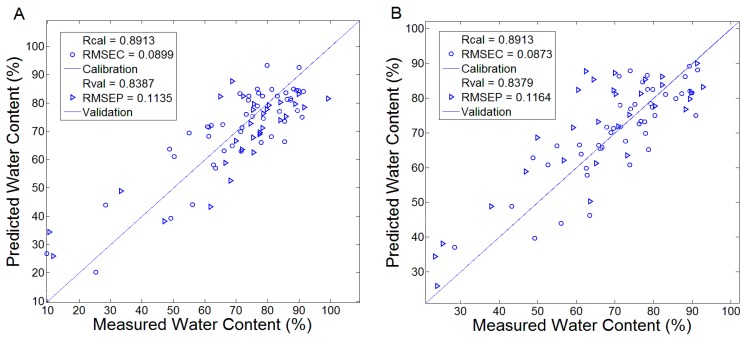
The calibration and validation results of PLS models for predicting WC. (**A**) Established by the transmission spectra; (**B**) Established by the absorption spectra.

The PLS method was performed to evaluate the correlation between the spectral data and leaf WC. The modeling results of the calibration set and validation set were discussed. A total of 5 principal components were determined to establish these PLS models. Figure 4A,B plotted the results that were modeled by the transmission spectra and the absorption spectra, respectively. The Rcal and RMSEC of the PLS models based on transmission and absorption spectra were (0.8913, 0.0899) and (0.8913, 0.0873), respectively. The Rval and RMSEP of these two PLS models were (0.8387, 0.1135) and (0.8379, 0.1164), respectively. As shown, the similar modeling results were obtained by using transmission and absorption spectra, which reflected the effectiveness and stability of PLS in dealing with these two kinds of spectra. Furthermore, the models all achieved reliable calibration and validation results. It implied THz transmission and absorption spectra in the range of 0.3–2 THz can quantitatively analyze leaf WC. 

#### 3.3.2. Predicting Leaf Water Content with KPLS Model

**Figure 5:**
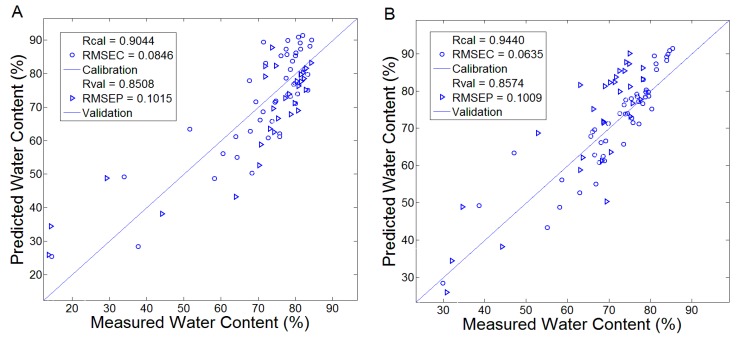
The calibration and validation results for WC prediction using the KPLS models. (**A**) Established by the transmission spectra; (**B**) Established by the absorption spectra.

KPLS was performed using the same calibration and validation data as PLS. The radial basis functions (RBF) were chosen as the kernel functions, and 2 principal factors were determined to establish the KPLS models. Figure 5 plotted the measured and predicted leaf WC by KPLS models based on the transmission and absorption spectra in the range of 0.3–2 THz. The Rcal and RMSEC of the KPLS models based on transmission and absorption spectra were (0.9044, 0.0846) and (0.9440, 0.0635), respectively. The Rval and RMSEP of the KPLS models were (0.8508, 0.1015) and (0.8574, 0.1009), respectively. The modeling performance based on the absorption spectra was better than that based on the transmission spectra. The reason was that the absorption spectral data exhibited nonlinear characteristics in the range of 0.3–2 THz. It increased the spectral specificity and improved the modeling performance of KPLS. By comparing with PLS, KPLS obtained better modeling performance. Due to its powerful data processing capability, it projected the original data onto higher dimensional feature space with kernel function. 

#### 3.3.3. Predicting Leaf Water Content with Boosting-PLS Model

Boosting-PLS was based on the concept of ensemble strategy. It built a series of PLS base models. The final modeling results were the combination of the results calculated by the established PLS base models. The number of iterations was set as 10, 20, 30, 40, 50, respectively, to test the effect of the number of iterations on modeling performance. Table 2 listed the calibration and validation results of the Boosting-PLS models with different iterations. In the iterative process of Boosting-PLS, each PLS base model was established with different sample set conducted by re-sampling. Hence, a certain degree of randomness was introduced into the Boosting-PLS model. Different results were obtained in different executions. For each number of iterations, the model was repeated 10 times and the mean values were taken as the final results of Boosting-PLS. As shown in Table 2, the mean values of the modeling results were reliable and stable with different number of iterations. In addition, the standard deviations were relatively small, which reflected the stability of Boosting-PLS. PLS and KPLS used the full calibration set to establish the models. Comparatively, Boosting-PLS used different subsets of the calibration set to establish the base models in the iterative process. Boosting-PLS obtained moderate results. The mean modeling results of Boosting-PLS under 5 different iterations were calculated. The mean Rcal and RMSEC of the models based on transmission and absorption were (0.8590, 0.1382) and (0.8617, 0.1386), respectively. The mean Rval and RMSEP of that were (0.8475, 0.1611) and (0.8472, 0.1376), respectively. According to these mean results of statistics, the modeling performance of Boosting-PLS was generally better than PLS but worse than KPLS. Boosting-PLS applied the ensemble strategy to adequately excavate the information from the calibration set. It was an integrated model based on PLS and it had better data processing capability than PLS. However, the data processing of Boosting-PLS was still in low dimensional linear space, while KPLS projected data to high dimensional nonlinear space. In general, all these three kinds of models achieved reliable results for predicting leaf WC. 

In this study, the THz transmission and absorption spectra in the range of 0.3–2 THz combined with PLS, KPLS, and Boosting-PLS modeling methods were applied for detecting WC in rapeseed leaves. KPLS model that was established based on absorption spectra produced the most accurate model with Rval = 0.8574 and RMSEP = 0.1009. In the previous works, the spectroscopy methods including visible, near and short-wave-infrared spectroscopy [28], mid to thermal infrared spectra [29], automated high throughput RGB and hyperspectral imaging [30,31], were applied as the detection technologies. Generally, for these methods, the characteristic wavelength need to be extracted otherwise a large number of leaf water indexes calculated by the mathematical operations of the specific wavelengths need to be explored. THz spectroscopy was sensitive to leaf water and the spectral processing was relatively simple [32]. Furthermore, THz spectroscopy could be analyzed by using the common modeling methods including continuous wavelet analysis [33], spectroscopic continuum removal and PLS [34]. The continuous wavelet analysis method was applied to calculate the function of wavelength for multiple dyadic scales, and a series of linear regression models were examined to predict leaf WC. The results were good but the algorithm was complex. For the spectroscopic continuum removal and PLS models, the algorithm was simple but the results need to be improved. The methods of KPLS and Boosting-PLS applied in this study were simple as well as had better data processing capability and modeling performances than that of PLS. Through these comprehensive comparison analysis, the results showed that the application of THz spectra with the KPLS and Boosting-PLS modeling methods offered effective detection of leaf WC.

## 4. Conclusions

This research was conducted to detect the WC in rapeseed leaves using THz sensor. The measurements of spectra and WC of the same rapeseed leaves were conducted every half an hour, and 10 sets of data were measured continuously. In this process, the dehydration of leaf moisture resulted in a constant decrease in WC. The response of THz spectrum to the change of leaf WC was discussed. The change regulation of the WC could be obviously observed from the THz spectra. The THz transmission and absorption spectra in the range of 0.3–2 THz were obtained. The spectra showed that the lower leaf WC led to the higher transmittance and lower absorption coefficient. The modeling methods including PLS, KPLS, Boosting-PLS were applied to establish the prediction models for WC based on the THz transmission and absorption spectra. KPLS projected the original input data onto higher dimensional space, which had the ability to process nonlinear data and generate the best modeling performance. Boosting-PLS conducted the ensemble strategy to establish a series of base models with different sample subsets, which could fully excavate the information contained in the calibration set. As well, PLS achieved relatively reliable results for predicting leaf WC. The results showed that THz sensor provided valuable information for studying physiological activities and detecting WC of rapeseed leaves. Combining with THz sensor and the modeling methods, the WC in rapeseed leaves could be effectively predicted. The results provided a theoretical basis for detecting the leaf WC of plants. It showed prospect in the field of agriculture. 

## Figures and Tables

**Table 1:** Statistical results of sample sets.

Spectra	Sample Set	N ^a^	Range (%)	Mean (%)	SD
Transmission	Calibration	50	1.48–91.39	70.00	0.2002
	Validation	30	25.93–87.77	68.02	0.1597
Absorption	Calibration	50	1.48–91.39	68.09	0.1946
	Validation	30	25.93–90.04	71.20	0.1699
	Full set	80	1.48–91.39	69.25	0.1853

N ^a^ was number of samples. SD was standard deviation.

**Table 2:** The calibration and validation results for WC prediction using Boosting-PLS models.

N ^b^	Transmission				Absorption			
	Calibration		Validation		Calibration		Validation	
	Rcal	RMSEC	Rval	RMSEP	Rcal	RMSEC	Rval	RMSEP
10	0.8599	0.1371	0.8497	0.1573	0.8581	0.1359	0.8466	0.1413
20	0.8578	0.1812	0.8479	0.2070	0.8639	0.1300	0.8455	0.1362
30	0.8598	0.1416	0.8481	0.1614	0.8656	0.1287	0.8471	0.1224
40	0.8567	0.1174	0.8464	0.1428	0.8565	0.1254	0.8497	0.1249
50	0.8606	0.1137	0.8453	0.1371	0.8645	0.1641	0.8472	0.1632

N ^b^ was the number of iterations. Rcal and Rval were the correlation coefficients of the calibration set and validation set, respectively. RMSEC and RMSEP were the root mean errors of the calibration set and validation set, respectively.
